# Association of Prenatal Healthy and Unhealthy Plant-Based Diets with Behavioral Outcomes in Preschool Children

**DOI:** 10.3390/nu17081372

**Published:** 2025-04-18

**Authors:** Esther Cendra-Duarte, Nerea Becerra-Tomás, Josefa Canals, Cristina Jardí, Victoria Arija

**Affiliations:** 1 Universitat Rovira i Virgili, Nutrition and Mental Health (NUTRISAM) Research Group, 43204 Reus, Spain; ecendra.tgn.ics@gencat.cat (E.C.-D.); nerea.becerra@urv.cat (N.B.-T.); josefa.canals@urv.cat (J.C.); cristina.jardi@urv.cat (C.J.); 2 Collaborative Group on Lifestyles, Nutrition, and Tobacco (CENIT), Institut d’Investigació en Atenció Primària IDIAP Jordi Gol, Institut Català de la Salut (ICS), 43202 Reus, Spain; 3 Institut d’Investigació Sanitària Pere Virgili (IISPV), 43003 Tarragona, Spain; 4 Universitat Rovira i Virgili, Department of Psychology, Centre de Recerca en Avaluació i Mesura de la Conducta (CRAMC), 43007 Tarragona, Spain

**Keywords:** plant-based diets, pregnancy, maternal diet, child, child behavior, neurodevelopment

## Abstract

Background/Objectives: Dietary patterns rich in plant-based foods during pregnancy have been associated with neurodevelopmental outcomes in offspring. However, not all components of these diets are healthy, and the impact of their quality on behavioral outcomes remains unexplored. Therefore, this study aimed to examine the association between healthy and unhealthy plant-based diets and offspring behavioral problems at the age of four. Methods: This research involved 201 mother–child pairs from the ECLIPSES study. Maternal diet during pregnancy was assessed using a validated food frequency questionnaire, from which the healthy plant-based diet index (hPDI) was calculated, emphasizing the consumption of fruits, vegetables, legumes and nuts, along with the unhealthy plant-based diet index (uPDI), highlighting the intake of sugary drinks and refined grains. Children’s behavior was evaluated using the Child Behavior Checklist 1.5-5. Multivariable logistic regression analyses were conducted to estimated odds ratios (ORs) and their 95% confidence intervals (CIs). Results: Greater adherence to the uPDI during pregnancy was associated with higher odds for externalizing problems, including attention-deficit/hyperactivity problems (OR = 1.08; 95%CI from 1.01 to 1.16) and oppositional defiant behavior (OR = 1.09; 95%CI from 1.00 to 1.19) in offspring, particularly girls. Higher adherence to the hPDI was not associated with children’s behavior. Conclusions: The consumption of unhealthy components of a plant-based dietary pattern during gestation has been associated with adverse behavioral outcomes in children at age four. These findings underscore the importance of discerning between the healthy and unhealthy components of plant-based diets when assessing their impact on child development.

## 1. Introduction

Maternal diet during pregnancy exerts a significant influence on fetal neurodevelopment by supplying essential nutrients to the fetus via placental transfer, which are required for a proper brain development [1]. An impairment in this process may lead to complications in childhood, including cognitive and behavioral difficulties that can interfere with the child’s functioning and overall development [2].

In recent years, plant-based diets have gained popularity [3], with an increasing number of pregnant women adopting this dietary pattern [4]. Although these diets primarily emphasize the consumption of plant-derived foods (i.e., vegetables, fruits, nuts, seeds, legumes and whole grains), there is no universally accepted definition of what constitutes a plant-based diet. Certain plant-based dietary patterns include limited amounts of animal-based foods, such as the Mediterranean diet (which includes fish [5]) or vegetarian diets (which can include eggs and dairy products [6]), while others exclude animal products entirely, such as vegan diets [6].

Previous studies examining the Mediterranean diet during pregnancy have reported favorable association with offspring cognition and behavioral outcomes [7,8]. Furthermore, other studies have primarily focused on comparing vegan and vegetarian diets with omnivorous diets, finding that plant-based diets were related to adverse anthropometric results, including an increased risk of smaller neonatal size and lower birth weight [4,9,10], while no associations have been observed with cognitive development [11]. These results may be attributed to the fact that not all dietary patterns rich in plant-based foods are inherently healthy, as not every plant-derived food provides the same nutritional benefits [12]. For instance, sugar-sweetened beverages and refined grains are technically classified as plant-based foods, but their nutritional profile is notably deficient. The increasing availability of ultra-processed non-animal or meat-alternative products high in sugars, salt, saturated fats and refined ingredients introduces lower-quality dietary options and variability in the health effects of plant-based diets [13,14]. In contrast, a well-balanced plant-based diet prioritizes nutrient-dense foods that are rich in vitamins, antioxidants, minerals and bioactive compounds, which are more effective in improving health [15]. In fact, to differentiate plant-based foods based on their health effects, and thereby capture the nutritional quality of plant-based diets, two distinct indices have been developed: the healthy plant-based diet index (hPDI) and unhealthy plant-based diet index (uPDI) [16].

In this context, with a lack of research evaluating the quality of plant-based dietary patterns during gestation and their impact on children’s behavior, the present study sought to evaluate the association of healthy and unhealthy plant-based diets during pregnancy and their children’s behavior at the age of four years in a cohort from the Mediterranean region of Spain. Furthermore, we also aimed to investigate whether the observed associations could vary depending on the child’s sex.

## 2. Materials and Methods

### 2.1. Study Population

**Figure 1:**
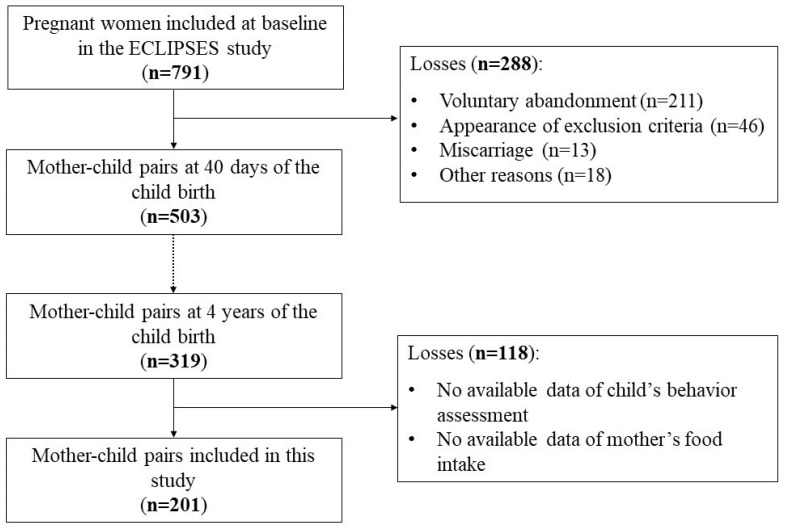
Flowchart of the study population.

The present study is based on data from the ECLIPSES study, a community-based randomized trial conducted among pregnant women in Tarragona, Catalonia, Spain [17]. The objective of this study was to evaluate the effect of iron supplementation during pregnancy on maternal and child-related outcomes. The participants were recruited during routine prenatal visits at primary care centers and selected based on specific inclusion criteria: women must be aged over 18 years, in their first 12 weeks of pregnancy, have no anemia, and be able to understand either of the official languages (Catalan or Spanish) as well as the study’s procedures. This analysis employs a subsample of 201 mother–child pairings with available data on maternal dietary food intake during gestation and behavioral assessments of the child at age four (Figure 1).

### 2.2. Assessment of Dietary Intake and Plant-Based Diet Indices

Maternal dietary intake during pregnancy was evaluated through the administration of a validated semi-quantitative Food Frequency Questionnaire (FFQ) [18], which participants completed by self-reporting. The questionnaire comprised 45 items, which participants were asked to complete at weeks 12, 24, and 36 of pregnancy, indicating their weekly or monthly intake of each item. Daily consumption for each item in grams/day was estimated by multiplying the reported consumption frequency by the average daily intake [19]. Total daily energy intake was calculated using the REGAL (Répertoire Général des Aliments) food composition table [20], and data from the Mataix Verdú Spanish food composition table [21].

The methodology proposed by Satija et al. [16] was employed to construct two plant-based diet indices: the hPDI and the uPDI. The indices comprise 14 food groups established based on their nutritional and culinary similarities, classified as either healthy or less-healthy plant-based foods, along with animal-based foods (Table 1). The consumption of each food group was categorized into quintiles, with the lowest consumers assigned to the lowest quintile and the highest consumers assigned to the highest quintile. Quintiles were assigned scores on a scale of 1 to 5 for each diet index. For the hPDI, healthy plant food groups were assigned a positive score, with quintile 1 receiving a score of 1 and quintile 5 receiving a score of 5, while less healthy food groups were reverse scored (quintile 1 = score 5, quintile 5 = score 1). In contrast, for the uPDI, less healthy plant food groups were assigned a positive score, while healthy food groups were reverse scored. The scoring of animal food groups was reversed for the two indices. The indices were calculated by summing the scores of the 14 food groups for each individual. The total score for each index, which ranged from 14 to 70, reflected the participant’s adherence to the specific plant-based dietary pattern.

### 2.3. Child Behavioral Problems

The Child Behavior Checklist for children aged 1.5 to 5 years (CBCL 1.5-5) was employed to evaluate behavioral problems, with responses provided by parents [22]. The tool comprises 99 items, evaluated on a three-point scale (not true, sometimes true, very true), which reflect parental perceptions of the child’s behavioral, emotional and social problems. The assessment generates six syndrome scales (emotional reactivity, anxious/depressed, somatic complaints, withdrawn, attention problems, and aggressive behavior), along with five DSM (Diagnostic and Statistical Manual of Mental Disorders)-oriented scales [depressive problems, anxiety problems, autism spectrum disorder problems, attention-deficit/hyperactivity disorder (ADHD) problems, and oppositional defiant disorder (ODD) problems]. These scales contribute to three broad categories: internalizing problems (including emotional reactivity, being anxious/depressed, somatic complaints, and being withdrawn), externalizing problems (covering aggressive behavior and attention problems), and total problems, which includes all syndrome scales. T-scores are employed for the interpretation of results, with scores of 65–69 classified as borderline, while those equal to or above 70, falling within the clinical range for syndrome and DSM-oriented scales. In the case of broad-band scales, scores between 60 and 64 indicate a borderline range, while scores of 65 and above indicate a clinical range. For the purpose of analyzing categorical data, scores above the borderline range were grouped as clinical scores. The Spanish validation of the CBCL 1.5-5 demonstrates strong internal consistency and effective discrimination in the identification of both disruptive and internalizing disorders in Spanish preschool-aged children [23].

### 2.4. Assessment of Other Variables

The data collected from pregnant women at baseline included the maternal age, socioeconomic status (grouped as low, middle or high) calculated by occupational status using the Catalan classification of occupations (CCO-2011) [24], and general lifestyle factors such as smoking habits, evaluated through the Fagerström test [25], and alcohol consumption quantified by the FFQ [18]. At each trimester of pregnancy, the following data were recorded: maternal height and weight, to calculate the body mass index (BMI), physical activity, determined by the short version of the International Physical Activity Questionnaire [26], and anxiety levels assessed with the State-Trait Anxiety Inventory [27], with particular focus on trait anxiety scores in the subsequent analyses. Moreover, at the time of birth, the gestational age, the children’s sex, the children’s birth weight and the quality of the maternal and child attachment, assessed by the Parenting Stress Index [28,29] were collected. At the four-year assessment, data were gathered on the duration of breastfeeding and the quality of children’s diets, which was evaluated according to the Spanish Diet Quality Index [30], with a previous dietary assessment using a validated short food frequency questionnaire for children [31]. Moreover, the mothers completed the Spanish-validated version [32] of the Goldberg Anxiety and Depression Scale [33], which was employed to evaluate their emotional health, with a particular focus on symptoms of anxiety and depression.

### 2.5. Statistical Analyses

The descriptive statistics for the study are presented as follows: for continuous variables, the mean and standard deviation are reported; for categorical variables, the frequency and percentage are presented. The normality of continuous data were evaluated using the Shapiro–Wilk test. The two dietary indices were energy-adjusted using the residual method [34] and treated as continuous variables in the models. Multivariable logistic regression analyses were conducted to estimate the adjusted odds ratios (ORs) and 95% confidence intervals (CIs) for each behavioral problem relative to the adherence to the plant-based dietary indices. The models were adjusted for several potentially confounding factors: maternal age (years), maternal first trimester BMI (kg/m^2^), socioeconomic status (low-middle or high), anxiety during gestation (score), smoking during gestation (non-smoker/ex-smoker or current smoker), energy intake during gestation (kcal/day), physical activity during gestation (METs/min/week), intervention group (iron doses of 40 mg vs. 80 mg or 40 mg vs. 20 mg), gestational age (weeks), breastfeeding duration (months), maternal-child attachment (score), mother’s anxiety and depression at the four-year follow-up (yes or no), child’s sex (boy or girl), and child diet quality (score). Additionally, multivariable logistic regression analyses stratified by the sex of the children were performed, adjusting for the same confounding factors except for the child’s sex. The following confounders had missing values: maternal anxiety (4.97%), breastfeeding duration (2.98%), maternal and child attachment (1.49%), children’s diet quality (1.99%), maternal anxiety or depression at fourth year visit (0.99%) and child’s birth weight (3.48%). Missing data were imputed using the median value for quantitative variables and the most frequent category for qualitative variables [35].

For the statistical analyses, the significance level was set at *p* < 0.05. These analyses were conducted with IBM SPSS Statistics, version 29.0 (Armonk, NY, USA: IBM Corp).

## 3. Results

The general characteristics of the population included in this study are presented in Table 2. The mean age of the pregnant women was 31.8 ± 4.4 years, and their mean BMI was 25.0 ± 4.6 kg/m^2^. Nearly 25% had a high socioeconomic status and more than 15% smoked during pregnancy. They were moderately physically active and had a mean energy intake of 1747.5 ± 294.2 kilocalories per day. During the perinatal period, mothers had a low mean anxiety score, indicating low levels of anxiety symptoms. However, when the child was four years old, 62.7% of the mothers reported the existence of anxiety and depression symptoms. The mean score of the mothers for the hPDI was 42.0 ± 4.6, with 40.3% of them having medium adherence, while the mean score for the uPDI was 42.0 ± 6.4, with 36.3% of them exhibiting high adherence. The mean gestational age at birth was around 39.5 weeks and the mean age of the children was 4.3 ± 0.3 years, with 50.7% being girls. The mean scores for all CBCL 1.5-5 scales fell within the non-clinical range for both girls and boys.

**Figure 2:**
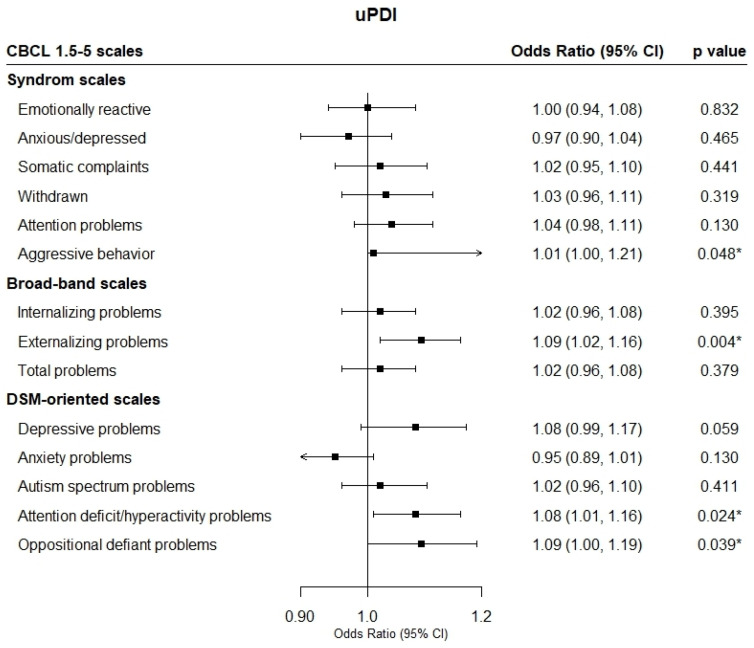

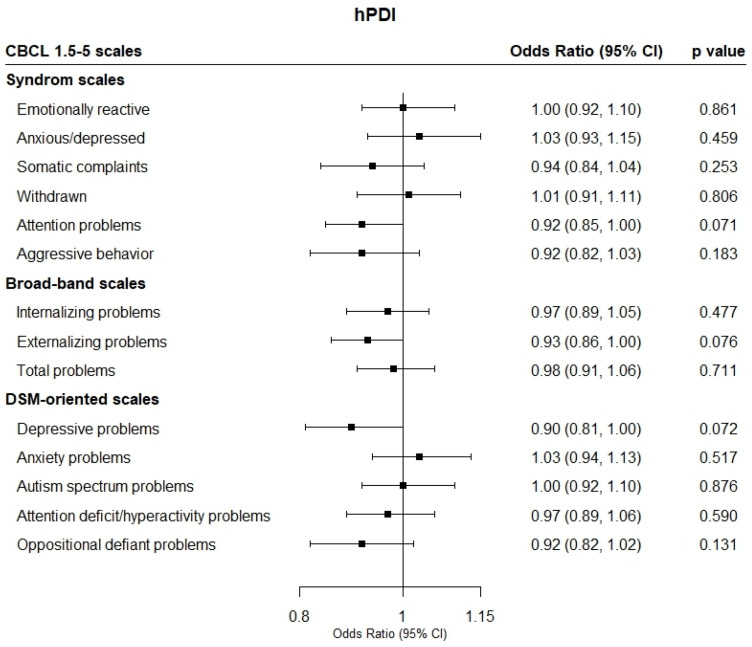
Multivariable-adjusted odds ratio of behavioral problems of children and maternal adherence to healthy (hPDI) and unhealthy (uPDI) plant-based diet indices. CBCL 1.5-5, Child Behavior Checklist 1.5-5; CI, confidence interval. Models were adjusted for maternal age, maternal first trimester body mass index, socioeconomic status, maternal smoking, anxiety during gestation, energy intake during gestation, physical activity, intervention group, gestational age, breastfeeding duration, maternal and child attachment, maternal anxiety or depression at the 4-year visit, child’s sex, and child’s diet quality. The diamonds indicate the odds ratios, while the whisker plots represent the 95% CIs. * *p*-value < 0.05.

Figure 2 presents the odds ratios (ORs) and 95% confidence intervals (CIs) for child behavioral problems in relation to maternal adherence to the two plant-based diet indices during pregnancy. The uPDI was found to be statistically significantly related, with increased ORs of having clinical scores for externalizing problems (OR = 1.09; 95% CI from 1.02 to 1.16), including aggressive behavior (OR = 1.01; 95% CI from 1.00 to 1.21), ADHD problems (OR = 1.08; 95% CI from 1.01 to 1.16), and ODD problems (OR = 1.09; 95% CI from 1.00 to 1.19) in children. No associations were found for the hPDI.

Table 3 shows the ORs for the association between maternal plant-based diet index scores and behavioral problems, stratified by child’s sex. Notably, higher maternal adherence to uPDI during gestation was related to higher odds for externalizing problems (OR = 1.18; 95% CI from 1.04 to 1.33) and attention-deficit/hyperactivity problems (OR = 1.20; 95% CI from 1.02 to 1.40) in girls, while in boys it was associated with lower odds for anxiety problems. However, no significant findings were observed for the hPDI in either boys or girls.

## 4. Discussion

In this population of pregnant Mediterranean women, greater maternal uPDI scores were associated with increased odds of offspring behavioral problems, specifically externalizing and ADHD problems, and particularly in girls. The influence of prenatal adherence to healthy or unhealthy plant-based dietary patterns on offspring outcomes has not been previously explored, highlighting the significance of this analysis.

In this study, the uPDI mainly accounts for plant-based foods with high carbohydrate content, especially sugar. Although glucose derived from carbohydrates is the primary source of energy for the mother and the fetal brain [36], excessive consumption of sugars during pregnancy may result in metabolic complications and neurobehavioral disorders in offspring [37]. Previous studies have identified a positive association between increased drinking of sugar-sweetened beverages during gestation and an elevated risk of delayed social–emotional development in offspring at 6–12 months of age [38] and ADHD symptoms at age 8 years [39]. In fact, a study conducted within our population determined that increased maternal adherence to a diet characterized by consumption of sugary foods and beverages, and superfluous animal products was related with emotional and externalizing behavioral problems in children at 4 years, also particularly in girls [40]. In animal models, higher maternal sucrose and fructose intake has been associated with greater impulsivity and hyperactive behavior, along with decreased attention in mice [41].

Carbohydrate intake significantly influences blood sugar levels and excessive consumption can lead to the onset of hyperglycemia [36]. During gestation, hyperglycemia has been related to a higher risk of externalizing problems in children at ages three and five [42]. Additionally, complications such as obesity and gestational diabetes, linked to glucose dysregulation, have also been tied to adverse neurobehavioral outcomes [43,44]. Elevated sugar levels can trigger adverse processes, including oxidative stress, hyperinsulinemia, iron deficiency, systemic inflammation, and insulin resistance, all of which may impair fetal brain development and contributing to neurodevelopmental dysregulation by disrupting central nervous system maturation [44] and inducing hippocampal neuronal alterations [45,46]. Moreover, maternal glucose and fructose can cross the placenta, influencing fetal development through alterations in gene expression and the regulation of glucose homeostasis [47]. Therefore, it may be advisable to encourage pregnant women to prioritize the quality of plant-based foods, ensuring to avoid processed foods, which typically contain non-nutritious components, in order to minimize potential developmental risks for the offspring.

Our results also showed that higher maternal adherence to the uPDI was associated with a greater likelihood of externalizing and ADHD problems in girls. Previous research on animals has indicated that elevated sugar intake (fructose and sucrose mainly) during gestation may exert sex-specific effects on fetal development outcomes, including growth factors [48] and metabolism [49,50,51,52]. On humans, a prior study in our population also observed this difference on behavioral outcomes [40]. These studies have consistently observed that the impact was more pronounced in girls, suggesting a heightened susceptibility to excessive maternal sugar intake [48,51]. Moreover, Solberg et al. [53] found that insufficient maternal fiber intake during pregnancy could elevate ADHD symptom levels in children, and this association was also found more prone in girls. Some of the mechanisms that may explain this sex difference include variations in DNA methylation and gene expression during pregnancy. Female fetuses have higher expression of key epigenetic regulators, influenced by chromosomal and hormonal factors, and are therefore more vulnerable to nutritional deficiencies [54,55]. Nevertheless, further studies are necessary to elucidate the exact underlying mechanisms of this difference.

The present study did not find an association between adherence to the hPDI during pregnancy and behavioral problems. A possible explanation for this could be that these diets may not provide essential nutrients typically derived from animal protein sources. A healthy plant-based diet, although rich in folic acid, antioxidants, phytochemicals, and carotenoids, tends to be lower in other important nutrients such as vitamin B12, iodine, and omega-3 fatty acids [56,57,58], which are primarily found in diets that include a moderate intake of animal products. They also play an important role in fetal neurodevelopment as the proper formation and maturation of the fetal brain are contingent upon an adequate provision of these essential compounds [11,58]. For instance, prior research in our population has observed a protective association between the Mediterranean diet, which incorporates animal-derived protein sources rich in these nutrients, and behavioral problems [8]. In fact, the Academy of Nutrition and Dietetics considers that, if properly planned, vegetarian and vegan diets might be appropriate in different life stages, including, also, the pregnancy period [59].

This study possesses several strengths and limitations that must be taken into account when interpreting the results. Firstly, this research benefits from having a sample drawn from a long-term intervention study with extended follow-up, which allowed for the collection of a wide range of data and variables to support detailed analyses. Nonetheless, despite the adjustment for various potential confounders, the observational design of the study cannot entirely rule out the possibility of residual confounding, including genetic predisposition. Secondly, our results may be limited to geographical and sociocultural populations similar to ours, which restricts their generalizability to other populations with different environmental backgrounds. Thirdly, recall bias and measurement error may be present in the dietary assessment obtained from the self-administered FFQ, which is usual in all studies using this method to evaluate dietary intake. However, the FFQ had been validated for our population and was administered to mothers during pregnancy, offering a broader perspective on their nutrition throughout the gestational period. Fourthly, the number of mother–child pairs who completed the assessments was reduced compared to the start of the original study due to a voluntary drop-out, which is common in long-term follow-up intervention studies. Fifthly, the children behavioral assessment relied on parent-reported data rather than clinical evaluation. Nonetheless, it was conducted using a well-established and validated tool in the field, which enhances the credibility and accuracy of the results. Finally, given the observational nature of this study, causality cannot be established.

## 5. Conclusions

In conclusion, adherence to a poor-quality plant-based dietary pattern during pregnancy may increase the likelihood of externalizing behavioral problems in preschool children, especially in girls. Therefore, our findings highlight the need to consider the quality of plant-based foods during pregnancy. Further research is needed to confirm our results in other populations with larger sample sizes and to explore the sex-specific mechanisms driving differences between boys and girls.

## Figures and Tables

**Table 1:** Classification of food groups and their components.

Food Groups	Foods Included	Scoring Criteria	
Healthy Plant Foods		hPDI	uPDI
Fruits	Fresh and preserved fruits	Q1 = 1 Q2 = 2 Q3 = 3 Q4 = 4 Q5 = 5	Q1 = 5 Q2 = 4 Q3 = 3 Q4 = 2 Q5 = 1
Vegetables	Lettuce, tomato, green beans, chard, spinach, eggplant, mushrooms, etc.		
Nuts	All types of nuts		
Legumes	Lentils, chickpeas, beans, etc.		
**Unhealthy Plant Foods**			
Juices	All types of fruit juices	Q1 = 5 Q2 = 4 Q3 = 3 Q4 = 2 Q5 = 1	Q1 = 1 Q2 = 2 Q3 = 3 Q4 = 4 Q5 = 5
Refined grains	Breakfast cereals, rice, pasta, bread, muffins, biscuit.		
Potatoes	Baked, fried or boiled potatoes and chips.		
Sugar-sweetened beverages	All types of sugar-sweetened beverages		
Sweets and desserts	Chocolate, chocolate bars, candies, cookies, doughnuts, croissants, cakes.		
**Animal Foods**			
Dairy products	Milk, yogurt, all types of cheese, custard, flan, ice cream.	Q1 = 5 Q2 = 4 Q3 = 3 Q4 = 2 Q5 = 1	Q1 = 5 Q2 = 4 Q3 = 3 Q4 = 2 Q5 = 1
Eggs	Eggs		
Fish and seafood	White fish (hake, grouper, sole, cod, etc.), oily fish (sardines, tuna, salmon, etc.) and other seafood (mussels, shrimp, prawns, squid, etc.)		
Meat	Chicken, turkey, beef, pork, lamb, minced beef, ham, sausage, hamburger.		
Other animal-based foods	Soup, croquettes, pizza.		

hPDI: healthy plant-based diet index. uPDI: unhealthful plant-based diet index. Q: quintile. The consumption of food was categorized into quintiles (from Q1 to Q5) and assigned a score ranging from 1 to 5. In the hPDI, healthy plant foods received higher scores for high consumption, while unhealthy plant foods and animal foods received higher scores for low consumption. In the uPDI, unhealthy plant foods scored higher as high consumption and healthy plant foods and animal foods scored higher as less consumption.

**Table 2:** Characteristics of the participants.

*Sample size*, *n* = *201*		
***Maternal Characteristics***		
Age (years)	31.8 ± 4.4	
First trimester BMI (kg/m^2^)	25.0 ± 4.6	
Socioeconomic status		
Low-middle	151 (75.1)	
High	50 (24.9)	
Smoke during gestation (yes)	33 (16.4)	
Alcohol during gestation (yes)	3 (1.5)	
Physical activity during gestation (METs/min/week)	2204.6 ± 2472.8	
Energy intake during gestation (kcal/day)	1747.5 ± 294.2	
Anxiety during gestation (score)	14.0 ± 8.1	
Anxiety and depression at 4-year visit (yes)	126 (62.7)	
hPDI (score)	42.0 ± 4.6	
Low adherence	59 (29.4)	
Middle adherence	81 (40.3)	
High adherence	61 (30.3)	
uPDI (score)	42.0 ± 6.4	
Low adherence	68 (33.8)	
Middle adherence	60 (29.9)	
High adherence	73 (36.3)	
***Children Characteristics***		
Age (years)	4.3 ± 0.3	
Sex (girl)	102 (50.7)	
Birth weight (grams)	3308.2 ± 434.9	
Gestational age (weeks)	39.5 ± 1.6	
Breastfeeding duration (months)	11.0 ± 12.5	
Maternal and child attachment (score)	51.8 ± 5.3	
Diet quality (score)	61.5 ± 10.7	
Behavioral Assessment (CBCL 1.5-5) (score)		
	Girls (n = 102)	Boys (n = 99)
Emotionally reactive	56.7 ± 8.3	58.0 ± 9.6
Anxious/depressed	56.0 ± 7.6	56.3 ± 7.4
Somatic complaints	55.7 ± 6.9	55.2 ± 6.9
Withdrawn	56.9 ± 6.7	59.4 ± 8.6
Attention problems	55.7 ± 6.6	60.0 ± 7.2
Aggressive behavior	53.9 ± 5.6	57.3 ± 8.6
Internalizing problems	54.2 ± 12.0	55.9 ± 12.0
Externalizing problems	50.9 ± 9.6	56.4 ± 11.8
Total problems	52.8 ± 11.3	56.8 ± 12.9
Depressive problems	55.8 ± 6.3	57.6 ± 8.2
Anxiety problems	57.7 ± 8.3	57.3 ± 8.3
Autism spectrum problems	56.5 ± 6.6	58.8 ± 8.2
Attention deficit/hyperactivity problems	55.7 ± 7.0	59.1 ± 8.3
Oppositional defiant problems	53.8 ± 5.7	56.1 ± 7.7

The values are presented as mean ± standard deviation (SD) or as frequencies and percentages (%). BMI: body mass index; hPDI: healthful plant-based diet index; uPDI: unhealthful plant-based diet index; CBCL 1.5-5: Child Behavior Checklist 1.5-5; DSM: Diagnostic and Statistical Manual of Mental Disorders.

**Table 3:** Multivariable logistic regression between the maternal adherence to healthy and unhealthy plant-based diet indices and behavioral problems at 4 years of age, stratified by child’s sex.

	Girls (n = 102)				Boys (n = 99)			
	hPDI		uPDI		hPDI		uPDI	
CBCL 1.5-5 Scales	OR (95% CI)	*p* Value	OR (95% CI)	*p* Value	OR (95% CI)	*p* Value	OR (95% CI)	*p* Value
**Syndrome Scales**								
Emotionally reactive	0.98 (0.84, 1.15)	0.879	1.03 (0.92, 1.15)	0.537	1.03 (0.91, 1.17)	0.589	0.98 (0.88, 1.09)	0.750
Anxious/depressed	1.04 (0.89, 1.22)	0.577	1.02 (0.92, 1.14)	0.641	1.05 (0.90, 1.23)	0.464	0.91 (0.81, 1.03)	0.160
Somatic complaints	0.99 (0.83, 1.18)	0.956	1.10 (0.96, 1.26)	0.141	0.91 (0.76, 1.09)	0.335	0.97 (0.85, 1.10)	0.640
Withdrawn	1.04 (0.79, 1.37)	0.756	1.16 (0.95, 1.43)	0.134	0.99 (0.86, 1.13)	0.909	0.98 (0.89, 1.09)	0.814
Attention problems	0.86 (0.72, 1.03)	0.113	1.06 (0.95, 1.18)	0.263	0.91 (0.82, 1.03)	0.146	1.04 (0.95, 1.14)	0.358
Aggressive behavior	NE	NE	NE	NE	0.92 (0.79, 1.07)	0.322	1.07 (0.94, 1.22)	0.290
**Broad-band Scales**								
Internalizing problems	0.96 (0.84, 1.10)	0.632	1.02 (0.93, 1.11)	0.667	1.01 (0.90, 1.13)	0.820	1.02 (0.93, 1.12)	0.549
Externalizing problems	0.88 (0.76, 1.02)	0.104	1.18 (1.04, 1.33)	0.006 *	0.96 (0.86, 1.07)	0.483	1.04 (0.96, 1.14)	0.303
Total problems	0.96 (0.85, 1.09)	0.582	1.07 (0.97, 1.17)	0.136	1.00 (0.90, 1.12)	0.917	0.99 (0.90, 1.08)	0.875
**DSM-Oriented Scales**								
Depressive problems	0.80 (0.60, 1.06)	0.127	1.51 (0.98, 2.33)	0.060	0.91 (0.79, 1.05)	0.242	1.01 (0.91, 1.12)	0.761
Anxiety problems	0.99 (0.87, 1.13)	0.941	0.99 (0.91, 1.09)	0.959	1.17 (0.99, 1.39)	0.062	0.82 (0.72, 0.94)	0.007 *
Autism spectrum problems	0.96 (0.82, 1.12)	0.629	1.05 (0.95, 1.17)	0.304	1.04 (0.90, 1.19)	0.570	1.00 (0.89, 1.12)	0.981
Attention deficit/hyperactivity problems	0.99 (0.84, 1.16)	0.930	1.20 (1.02, 1.40)	0.022 *	0.96 (0.85, 1.08)	0.567	1.05 (0.95, 1.17)	0.268
Oppositional defiant problems	0.82 (0.63, 1.05)	0.128	1.24 (0.98, 1.56)	0.062	0.95 (0.82, 1.10)	0.507	1.06 (0.95, 1.19)	0.265

hPDI: healthful plant-based diet index. uPDI: unhealthful plant-based diet index. CBCL 1.5-5: Child Behavior Checklist 1.5-5. DSM: Diagnostic and Statistical Manual of Mental Disorders. OR: odds ratio. CI: confidence interval. Models were adjusted for maternal age, maternal first trimester body mass index, socioeconomic status, maternal smoking, anxiety during gestation, energy intake during gestation, physical activity, intervention group, gestational age, breastfeeding duration, maternal and child attachment, maternal anxiety or depression at the 4th year visit, child’s sex, and child’s diet quality. * *p*-value < 0.05. NE: value not estimable due to convergence errors in the model caused by a limited sample size or a lack of variability in one of the categories of the dependent variable.

## Data Availability

The data presented in this study are available on request from the corresponding author.

## Abbreviations

The following abbreviations are used in this manuscript:

ADHD	Attention-Deficit/Hyperactivity Disorder
BMI	Body Mass Index
DSM	Diagnostic and Statistical Manual of Mental Disorders
CBCL 1.5-5	Child Behavior Checklist 1.5-5
FFQ	Food Frequency Questionnaire
hPDI	Healthy Plant-based Diet Index
uPDI	Unhealthy Plant-based Diet Index
ODD	Oppositional Defiant Disorder
OR	Odds Ratios
CI	Confidence Intervals
